# Pembrolizumab-Induced Thyroiditis Shows PD-L1Expressing Histiocytes and Infiltrating T Cells in Thyroid Tissue - A Case Report

**DOI:** 10.3389/fimmu.2021.606056

**Published:** 2021-06-18

**Authors:** Jörg Jabkowski, Almute Loidl, Barbara Auinger, Helmut Kehrer, Norbert Sepp, Robert Pichler

**Affiliations:** ^1^ Department of Dermatology, Ordensklinikum Linz (Elisabethinen), Linz, Austria; ^2^ Institute of Pathology, Steyr Hospital, Steyr, Austria; ^3^ Department of Surgery, Steyr Hospital, Steyr, Austria; ^4^ Institute of Nuclear Medicine, Kepler University Hospital, Linz, Austria

**Keywords:** pembrolizumab, thyroiditis, melanoma, FDG, PET

## Abstract

**Context:**

Immune-related adverse events frequently take place after initiation of immune checkpoint inhibitors (ICI) therapy. The thyroid gland is the endocrine organ most commonly affected by ICI therapy, the pathological mechanism is still poorly understood.

**Case Description:**

A 60-year old Upper Austrian male melanoma patient under pembrolizumab therapy received thyroidectomy because of a suspicious FDG avid thyroid nodule. Histopathology showed a pattern comparable with thyroiditis de Quervain. The inflammatory process consisted predominantly of T lymphocytes with a dominance of CD4+ T helper cells. In addition CD68+ histiocytes co-expressing PD-L1 were observed.

**Conclusion:**

Clusters of perifollicular histiocytes expressing PD-L1 were observed in this case of pembrolizumab induced thyroiditis - probably induced by the former ICI therapy. This finding might indicate the initial target for the breakdown of self tolerance. In context with other data the immunological process seems to be driven by CD3+ lymphocytes infiltrating the thyroid.

## Case Report

Immunotherapy is a new effective therapeutic approach in oncology. Immune checkpoint inhibitors (ICI) are highly effective treatment of multiple malignancies refractory to conventional chemotherapies (1). Programmed cell death protein 1 (PD-1) inhibitors as pembrolizumab inhibit the immunological pathways that control T-cell activation or anergy and act to restore T cell mediated antitumor immunity (2). Immune-related adverse events (irAEs) frequently take place after initiation of checkpoint therapy. The thyroid gland is an endocrine organ frequently affected by ICI therapy. About one third of patients with ICI therapy develop thyroid dysfunction (3). The various forms of thyroid pathology induced on any ICI are presented by **Table 1**. Patients who develop thyroid irAEs show longer median survival than patients without, at least in lung cancer (8, 9). This indicates that the development of thyroid endocrinopathy might mirror the therapeutic goal of ICIs respective to cancer cells. Preexisting autoimmune thyroid disease is considered a risk factor, patients with known endocrine autoimmunity may have more frequent and severe irAEs with anti-PD-1 treatment (1). ICIs can induce worsening of autoimmune hypothyroidism, Graves´ disease (GD) hyperthyroidism and destructive thyroiditis (1, 10). De novo thyroiditis resembling (silent) thyroiditis de Quervain can occur even in constantly thyroid antibody negative persons, the pathological mechanism is still poorly understood.

**Table 1:** Immune related adverse effects of the thyroid caused by ICI therapy (4–7).

Molecular target	Pharmaceutical	Thyroid affection
CTLA-4-inhibitor	Ipilimumab	painless thyroiditis, thyroid storm
CTLA-4-inhibitor	Trememilumab	painless thyroiditis, thyroid storm
PD-1-antibody	Nivolumab	painless thyroiditis
PD-1-antibody	Pembrolizumab	painless thyroiditis
PD-1-antibody	Cemiplimab	hypothyroidism
PD-1-antibody	Spartalizumab	hypo- and hyperthyroidism
PD-L1-antibody	Atezolizumab	hypothyroidism
PD-L1-antibody	Durvalumab	hypothyroidism
PD-L1-antibody	Avelumab	hypothyroidism
NK-cell-based	Urelumab	not demonstrated
NK-cell-based	Relatlimab	not demonstrated
NK-cell-based	Linlumab	not demonstrated

## Clinical Presentation

**Figure 1:**
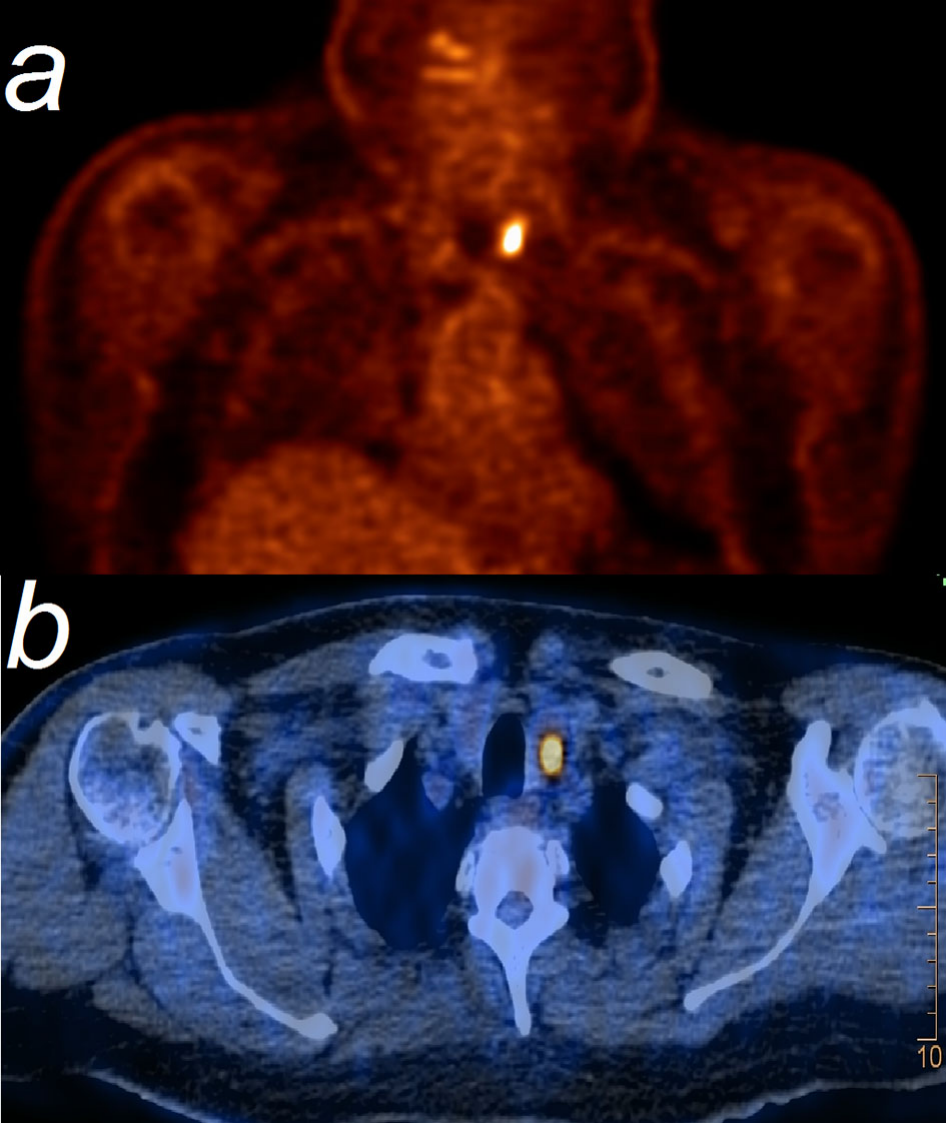
FDG-PET **(A)** shows a marked focal uptake with an SUV=14, corresponding to left thyroid lobe at PET/CT **(B)**.

A 60-year old Upper Austrian male presented at the institute of nuclear medicine in Steyr, Austria, for evaluation of possible thyroid malignancy. A FDG-PET/CT examination from March 2019 showed a marked focal uptake in the left thyroid lobe only (see **Figure 1**). The examination had taken place for restaging of melanoma. In August 2015 an exulcerated nodular melanoma (tumor thickness 3.5 mm pT3bN0M0 - stage IIB AJCC) had been operated on the right big toe. He had received adjuvant interferon therapy from August 2015 to January 2016. In February 2018 three lymph node metastases were surgically removed from the right groin, a complete lymph node dissection was not performed at this time. FDG-PET/CT in July 2018 verified a relapse in the right groin, another three lymph node metastases were histologically confirmed. Immunotherapy with a PD-1 antagonist was started, including eleven cycles of pembrolizumab therapy (240mg/cycle, every three weeks) until March 2019.

Thyroid examination in March 2019 revealed a nodular goitre with a volume of 20ml, the predominant and sonographically hypoechogenic nodule had a diameter of 17mm, was located in the left lobe and showed low uptake of Tc99m at scanning. TSH, fT4 and fT3 were in the normal range, TPO- and Tg-antibodies were negative. Palpation of the thyroid was not painful, sonography of the neck did not detect enlarged lymph nodes. Fatigue was the only clinical complaint.

Goitre is a frequent thyroid pathology in Austria, but the patient´s suspicious thyroid nodule was a new finding - as there had not been a thyroid examination before - and former PET scanning had not revealed any FDG avidity of the thyroid. Although retrospectively fine needle aspiration would have been indicated as the next diagnostic step, thyroidectomy was performed then in May 2019.

**Figure 2:**
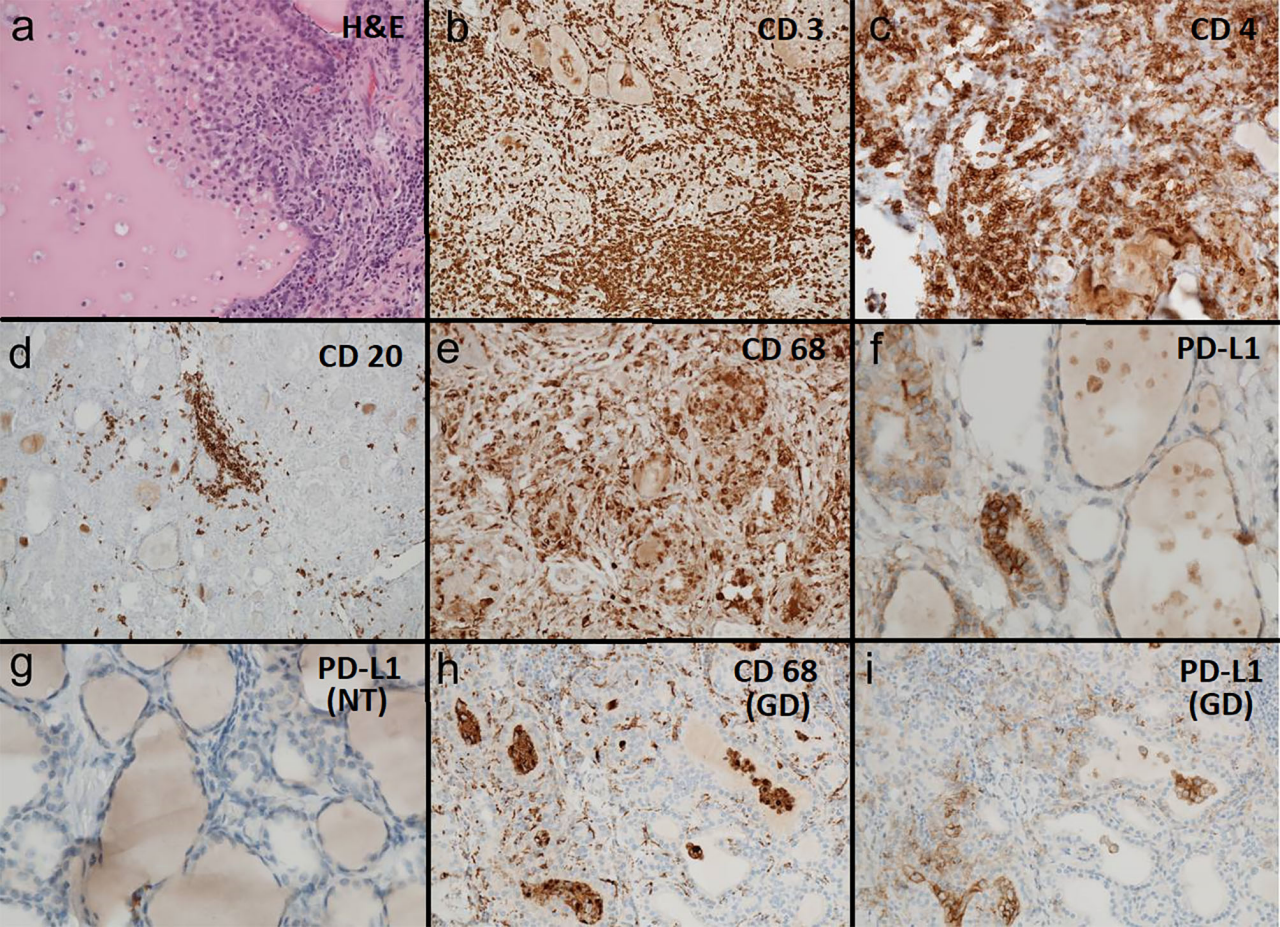
**(A–F)** Pembrolizumab induced thyroiditis: **(A)** H&E 200x: thyroid tissue with follicle partially destucted by histiocytes. **(B)** CD3 100x: thyroid tissue densely infiltrated by CD3 reactive T-lymphocytes. **(C)** CD 4 200x: thyroid tissue densely infiltrated by T helper cells (CD4 reactive). **(D)** CD 20 100x: thyroid tissue sparsely infiltrated by CD20 reactive B-lymphocytes. **(E)** CD 68 200x: thyroid tissue densely infiltrated and partially destructed by CD68 reactive histiocytes. **(F)** PD-L1 (SP263 hemalum2 8min) 400x: thyroid follicle partially destructed by histiocytes with coexpression of PD-L1 (clone SP263). **(G)** Regular thyroid tissue: PD-L1 (SP263 hemalum2 8min) 400x: normal thyroid tissue without PD-L1 (clone SP263) reactive cellular elements. **(H, I)** Graves´ Disease: **(H)** CD68 reactive histiocytes in the stroma and in thyroid follicles. **(I)** Histiocytes in the stroma and in thyroid follicles showing coexpression of PD-L1 (clone SP263).

Because of the medical history pembrolizumab induced thyroiditis was finally considered and evaluation of the immunological processes of the specimen was added (see **Figure 2**). Thyroiditis in our patient resembled thyroiditis de Quervain. No thyroid malignancy and obviously no cervical lymph node metastasis were detected. Histology of the presented case showed massive inflammation, superficially resembling granulomatous thyroiditis de Quervain. There was a marked parenchymal destruction with extensive fibrosis and scar tissue. The lymphocytic infiltrate consisted mostly of CD3 reactive T-cells, CD4 positive cells being somewhat dominant. No extensive dominance of CD8 reactive T-cells could be observed. No further immunohistological subtyping of the T-lymphocytes was performed. CD20 positive B-cells were clearly in the minority and could be seen only in little groups without any forming of follicles or germinal centers.

Only a few polyclonal CD138 reactive plasma cells were found. There was no increase of IgG4 expressing plasma cells.

The inflammatory infiltrate however consisted additionally of numerous CD68-reactive histiocytes being the second frequent population of inflammatory cells. Some epitheloid cell granulomas could be observed. Additionally pseudogranulomas were found due to destruction of thyroid follicles. Partly, in the center of the destructed follicles residual colloid was present.

Many histiocytes showed coexpression of PDL-1 being completely absent in not inflamed residual thyroid tissue.

Thyroid hormone therapy with 100µg levothyroxine daily was initiated after operation and re-evaluated in January 2020 when the patient presented in good clinical conditions. In respect to the melanoma repeated stagings in June, September and December 2019 with clinical dermatological examination, FDG-PET/CT, cranial MRI, lymph node sonography and serum measurement of s100 and LDH did not present any specific tumor manifestation.

## Discussion

Initiation of checkpoint therapy is often associated with irAEs (3). One third of patients treated with nivolumab develop irAEs of the thyroid (8).The pre-existence of autoimmune thyroid disease (AITD) is considered a risk factor for the development of ICI induced thyroiditis (11). Considering positive thyroid peroxidase (TPO) antibodies as a specific marker for AITD about 30-40% of patients with irAEs of the thyroid are part of this group (8, 11). This proportion is definitely higher than in the general population. On the other hand, the majority of patients develop ICI induced thyroiditis without AITD. TPOabs are usually not associated with ICI induced thyroid dysfunction (11). It has been speculated that in those cases undetected sero-negative AITD prior to initiation of ICI therapy might be existent (2).

(Diffuse) increased FDG uptake of the thyroid gland has been observed in 64% of patients with pembrolizumab induced thyroiditis (11), indicating the activity of the inflammatory process. This resembles our case, with the particular finding of a focal configured uptake in a suspicious nodule, which led to thyroidectomy. Increased FDG uptake is a well-known observation in any patient with AITD as well (12).

In AITD a destruction of the thyroid parenchyma due to an inflammatory infiltrate consisting of T-lymphocytes and plasma cells and variable forming of B-cell dominant polyclonal germinal centers can be observed.

The parenchyma shows microfollicular and trabecular arranged follicle cells with reduced colloid content. Typically multifocal oxyphylic metaplasia is to be found. Granulomas, histiocytes and giant cells are usually not present. A minority of AITD (10%) is of the fibrous variant. Therefore, our case presented a considerably different pattern (13).

In thyroiditis de Quervain, histiocytes, plasma cells and T lymphocytes dominate the active phase (13). Cytomorphical features of fine needle aspiration include many giant cells, which are considered histiocytes (14). Immune cells in the specimen of our patient consisted mostly of CD3+ T lymphocytes (predominantly CD4+ helper T-cells, about 10% CD8+ suppressor T-cells), while plasma T cells were only sparsely observed.

The PD-1/PD-L1 pathway is an important mechanism of peripheral tolerance (15). In flow cytometry of peripheral blood the fraction of PD-1+ CD4+ cells tends to be higher in GD than in healthy controls. In AITD glands two thirds of CD+ T lymphocytes express PD-1. Álvarez-Sierra et al. also showed that PD-L1 expression was observed in numerous thyroid follicles in most AITD patients. Therefore a specific target to break down immune tolerance in AITD under ICI could be assumed. On the contrary, 75% of tissue from non-autoimmune thyroids was negative for PD-L1, and none of them had a PD-L score >1 (15). Less than 10% of differentiated thyroid cancer cells show PD-L1 expression with a level of weak staining (16).

Our patient presented marked PD-L1 positivity in his thyroid gland focused in a patchy form at clusters of histiocytes. These images resemble that of a patient with GD we analysed for comparison (see **Figures 2H, I**) and also goes in line with a report of a patient with pembrolizumab induced thyroiditis in nodular goiter, where PD-L1 immunostaining highlighted predominantly histiocytes. We suppose this phenomenon to be induced by ICI therapy - as it cannot not be observed in regular multinodular goiter (15). PD-L1 is an inducible and highly dynamic receptor that can change over time. PD-L1 expression can be upregulated *via* interferon gamma *in vivo* (17). Thyroid follicular cells - even from non-autoimmune thyroid - exhibit PD-L1 expression in cell culture, which can be induced by interferon gamma (15). Melanoma patients with favourable response to ICI therapy have high expression of mRNA of interferon gamma (17), a condition which might correspond to our patient as well.

To our best knowledge, until now only one case report showed histological data of thyroid tissue under similar circumstances (10), so final conclusions are difficult to take. There is one more case report presenting the development of thyrotropin (TSH) receptor antibody (TRAb) positive GD under ipilimumab therapy which led to thyroidectomy. Immunohistochemistry was not done in this patient (18). Different to our case, the report of nivolumab induced thyroiditis showed a predominant CD8+ immune response - in proximity to the observed granulomas (19).

Published data about the role of immune cells in blood of patients with irAEs are available. Immunophenotyping by flow cytometry revealed that natural killer cells are elevated in both autoimmune and pembrolizumab induced thyroiditis patients (11). The role of different subsets of CD4+ T cells in tumor immunity - and autoimmunity - remains underappreciated, the plasticity of differentiation can be extensively modulated by the microenvironment (20). Baseline measurement before the occurrence of thyroid irAEs have shown higher levels of IL-2; IL-2 activated CD8+ T-cells are supposed to attack not only tumor but also thyroid cells (21). As a simultaneous immunological evaluation of peripheral blood samples was not done in our patient, a thoroughly contextual interpretation related to the histological findings is not possible. A predominant T cell modulated process can be assumed, anyhow. A recent investigation comparing fine needle aspiration data to blood analysis showed that PD-1 inhibitor induced thyroiditis had augmented CD4+PD1+ and CD8+PD1+ T lymphocytes in thyroid compared to being almost absent in blood (22). No single biomarker has proven to be sufficiently predictive for irAEs, but a relevant role of T-cell mediation has been demonstrated (23). Flow cytometry on blood samples of melanoma patients showed lower percentages of PD-1 expressing CD4+ and CD8+ T cells at baseline in patients with grade ≥3 irAEs after ICI therapy compared to those without (24).

## Conclusion

Thyroiditis is a frequent adverse event of ICI therapy. Preexisting AITD increases the risk but is not a requisite for irAEs of the thyroid. We present a case with the rare situation of consecutive thyroidectomy which enables pathohistological evaluation. It revealed clusters of perifollicular histiocytes expressing PD-L1 in the thyroid specimen - probably induced by former ICI therapy. This might indicate the initial target for the breakdown of self tolerance. In context with other data the immunological process seems to be driven by CD3+ lymphocytes infiltrating the thyroid.

## Data Availability Statement

The original contributions presented in the study are included in the article/**Supplementary Material**. Further inquiries can be directed to the corresponding author.

## Ethics Statement

Written informed consent was obtained from the individual(s) for the publication of any potentially identifiable images or data included in this article.

## Author Contributions

JJ and RP wrote the manuscript. AL prepared and interpreted the histologic specimens. BA was in charge of thyroid surgery and related process. HK and NS documented the management of melanoma. All authors contributed to the article and approved the submitted version.

## Conflict of Interest

The authors declare that the research was conducted in the absence of any commercial or financial relationships that could be construed as a potential conflict of interest.
