# Supplementary material for: Pembrolizumab-Induced Thyroiditis Shows PD-L1Expressing Histiocytes and Infiltrating T Cells in Thyroid Tissue - A Case Report
Source: Front Immunol. 2021 Jun 18;12:606056. doi: 10.3389/fimmu.2021.606056 (PMC8250423; doi:10.3389/fimmu.2021.606056)
Supplement: Supplementary file 1 [file DataSheet_1.doc]

**Materials and Methods: Antibodies for Immunohistochemistry**

| **Antibody** | **Clone** | **Dilution** | **Staining and Addenda** | **Producer** |
| --- | --- | --- | --- | --- |
| CD3 | 2GV6 | Ready to use | DAB (+ Amplification) | Ventana |
| CD4 | SP35 | Ready to use | DAB | Ventana |
| CD8 | C8/144B | Ready to use | DAB | Cell Marque |
| CD20 | L26 | 1:1000 | DAB | Dako |
| CD68 | KP-1 | Ready to use | DAB | Ventana |
| CD79 | SP18 | Ready to use | DAB | Ventana |
| CD138 | B-A38 | Ready to use | DAB | Cell Marque |
| IgG4 | MRQ-44 | Ready to use | DAB (+ Protease 1) | Ventana |
| Kappa | - (Kappa Light Chains Code No. A192) | 1:50 000 | DAB | Dako |
| Lambda | - (Lambda Light Chains Code No. A193) | 1:30 000 | DAB | Dako |
| PD-L1 | SP263 | Ready to use | Opti View DAB IHC | Ventana |
| TTF1 | SP141 | Ready to use | DAB (+Amplification) | Ventana |

**Further details:**

**Material:**

formalin-fixed-paraffin-embedded tissue - FFPE:

fixation: buffered formalin, 7,5% (SAV)

staining: hematoxilin-eosin-stain (H&E) (Merck – hematoxilin; hospital based pharmacy - eosin).

2µm sections for immunohistochemistry; 4µm sections H&E staining

**Platforms used for immunohistochemisty:**

Benchmark XT, Benchmark Ultra (Ventana, Roche)

**Staining and counterstaining of immunhistochemical antibody reaction:**

DAB (ultraView Universal DAB Detection Kit respective OptiView DAB IHC Detection Kit, both Ventana, Roche)

hemalum I+II (Ventana, Roche)
